# Pediatric radius torus fractures in x-rays—how computer vision could render lateral projections obsolete

**DOI:** 10.3389/fped.2022.1005099

**Published:** 2022-12-14

**Authors:** Michael Janisch, Georg Apfaltrer, Franko Hržić, Christoph Castellani, Barbara Mittl, Georg Singer, Franz Lindbichler, Alexander Pilhatsch, Erich Sorantin, Sebastian Tschauner

**Affiliations:** ^1^ Department of Radiology, Division of Neuroradiology, Vascular and Interventional Radiology, Medical University of Graz, Graz, Austria; ^2^ Department of Radiology, Division of Pediatric Radiology, Medical University of Graz, Graz, Austria; ^3^ Department of Computer Engineering, Center for Artificial Intelligence and Cybersecurity, University of Rijeka Faculty of Engineering, Rijeka, Croatia; ^4^ Department of Paediatric and Adolescent Surgery, Medical University of Graz, Graz, Austria

**Keywords:** wrist, fracture, radiography, artificial intelligence, radius

## Abstract

It is an indisputable dogma in extremity radiography to acquire x-ray studies in at least two complementary projections, which is also true for distal radius fractures in children. However, there is cautious hope that computer vision could enable breaking with this tradition in minor injuries, clinically lacking malalignment. We trained three different state-of-the-art convolutional neural networks (CNNs) on a dataset of 2,474 images: 1,237 images were posteroanterior (PA) pediatric wrist radiographs containing isolated distal radius torus fractures, and 1,237 images were normal controls without fractures. The task was to classify images into fractured and non-fractured. In total, 200 previously unseen images (100 per class) served as test set. CNN predictions reached area under the curves (AUCs) up to 98% [95% confidence interval (CI) 96.6%–99.5%], consistently exceeding human expert ratings (mean AUC 93.5%, 95% CI 89.9%–97.2%). Following training on larger data sets CNNs might be able to effectively rule out the presence of a distal radius fracture, enabling to consider foregoing the yet inevitable lateral projection in children. Built into the radiography workflow, such an algorithm could contribute to radiation hygiene and patient comfort.

## Introduction

In adults and children, the distal radius is the most common site for fractures. Injuries around the wrist are typically examined by digital radiography (DR). Standard DR procedures include two orthogonally-oriented projections of the wrist joint and the adjacent structures. Sometimes, additional projections are performed. The resulting images aggregate into a complete examination for interpretation by emergency physicians or radiologists.

Prior studies demonstrated that Artificial Intelligence (AI) is able to successfully detect fractures in radiographs (1–3). Radiological AI models usually originate from annotated image data, also known as supervised AI (4). With few exceptions, they fall into the domain of deep learning (DL) through convolutional neural networks (CNN) (5, 6). DL models commonly build upon large training image sets for robust outcomes (7, 8), often containing thousands, hundred-thousands, millions or more different samples. Corresponding radiological datasets (9) are typically magnitudes smaller because building and maintaining comprehensive DL data collections still is a major obstacle (7).

Buckle fractures—often found at the distal radius—represent a frequent type of incomplete fracture in pediatric patients. Although adequate therapeutic management remains a matter of debate, temporary immobilization of the affected wrist remains standard practice for patient comfort and prevention of secondary injury (10). Accurate detection of these, sometimes very subtle fractures can potentially be very challenging for human readers. We aimed to examine whether state-of-the-art CNNs are able to discriminate between incomplete distal metaphyseal radius fractures [AO classification (11) code “23r-M/2.1”] and normal bones in posteroanterior (PA) wrist radiographs of children. The main hypothesis was that AI could exceed human experts in recognizing these fractures in a single PA projection. We were also interested to find out, if there would be potential to forego the routinely acquired lateral x-ray projection through computer vision applications in the future.

## Materials and methods

We queried our local pediatric wrist radiography dataset (total number of images *n* = 20,330) for studies containing isolated incomplete metaphyseal distal radius fractures in PA projection (AO classification code “23r-M/2.1”). Overall, 1,337 radiographs fulfilled this criteria. All images had been acquired between 2008 and 2018. We matched the radiographs to normal PA wrist studies of patients with same age and sex, sampled from a pool of 2,641 images. As part of the matching procedure, two reference radiologists with 8 (S.T.) and 29 years (E.S.) of experience in pediatric trauma radiology revisited all negative cases, identified images in doubt for pathology, and replaced them if necessary. After this quality checking step, the dataset contained 1,337 pediatric PA wrist radiographs with, and 1,337 without isolated incomplete metaphyseal distal radius fracture.

Mean patient age of both groups (positive and negative for incomplete distal radius fractures) was 10.4 ± 3.3 years, ranging from 0 to 17 years of age. The final dataset (*n* = 1,337), as well as all sub-sets contained the same number of female and male patients.

**Figure 1:**
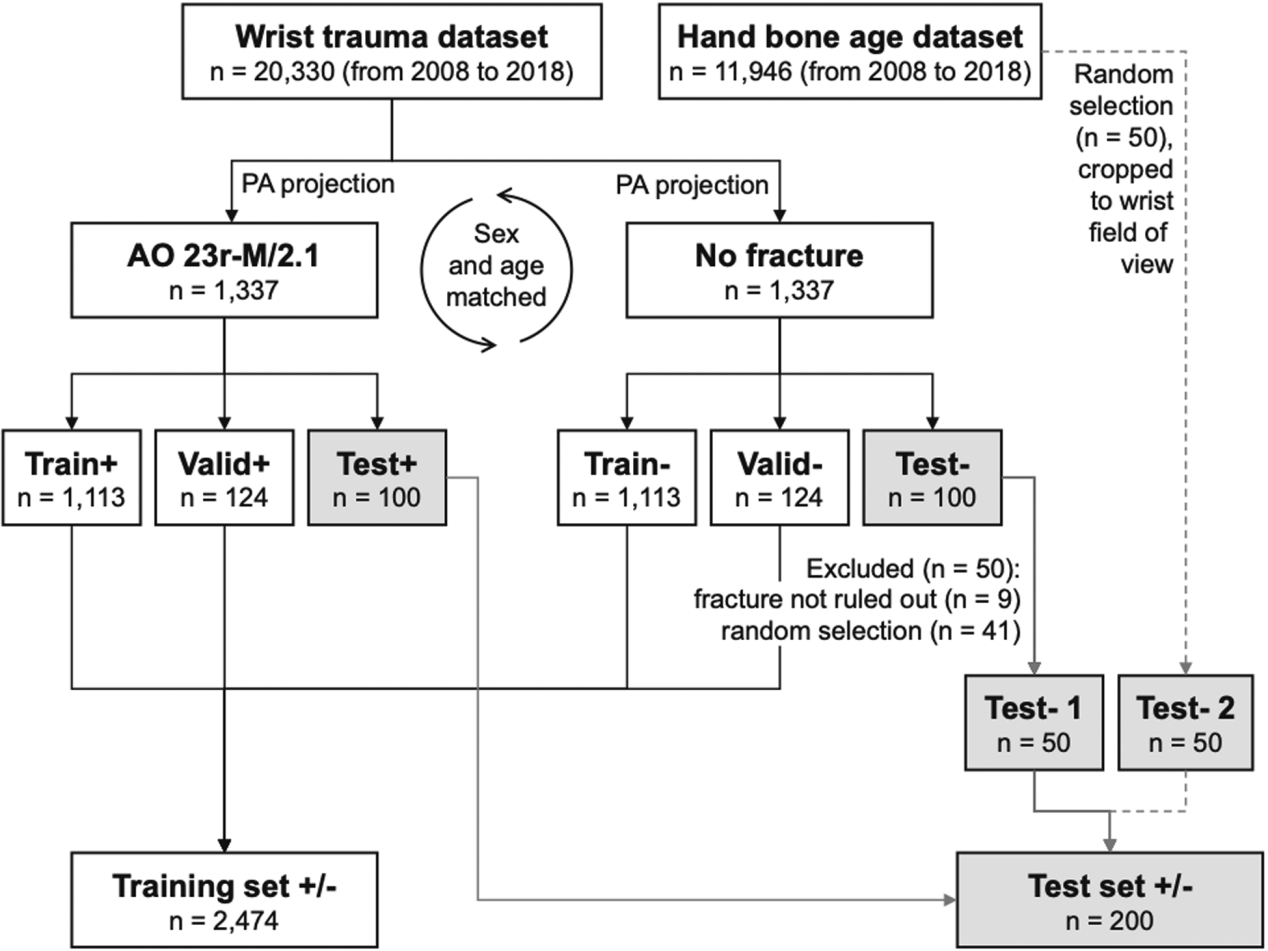
Flowchart depicting the datasets and the performed steps of obtaining training, validation, and testing subsets.

We randomly separated 100 images per group to serve as test set. The remaining 1,237 images were used for training CNN models, divided into 90% training data (*n* = 1,113 per group), and 10% validation data (*n* = 124 per group). As it is nearly impossible to definitely exclude a fracture in radiographs, particular attention was directed to the test set creation. Apart from the reference radiologists' readings, we decided to inject studies into the negative test set which almost never contain fractures, specifically hand bone age x-rays. The pool of these images was 11,946 acquired between 2008 and 2018. We decided to replace half of the negative wrist test set (*n* = 50) by a random selection of hand bone age radiographs, manually cropped to typical wrist fields of view. The replaced wrist radiographs were (1) manually chosen because fractures could not be ruled out entirely by the reference radiologists (*n* = 9); and (2) randomly selected to fill the residual number up to fifty cases (*n* = 41). Due to the random selection, test set age in negative cases was altered to 10.8 ± 3.7 years vs. 10.3 ± 3.4 years in positive test set radiographs. To summarize, as it is depicted in Figure 1, the training set contained 2,226 images, the validation set had 248 images, and the test set had 200 images.

### Image acquisition

Radiographs had been acquired on different computed (CR) or digital radiography (DR) equipment by radiological technologists of the Division for Pediatric Radiology, Department of Radiology, Medical University of Graz, Austria. The studies had been stored as Digital Imaging and Communications in Medicine (DICOM) images in the local Picture Archiving and Communication System (PACS).

### Image processing

**Figure 2:**
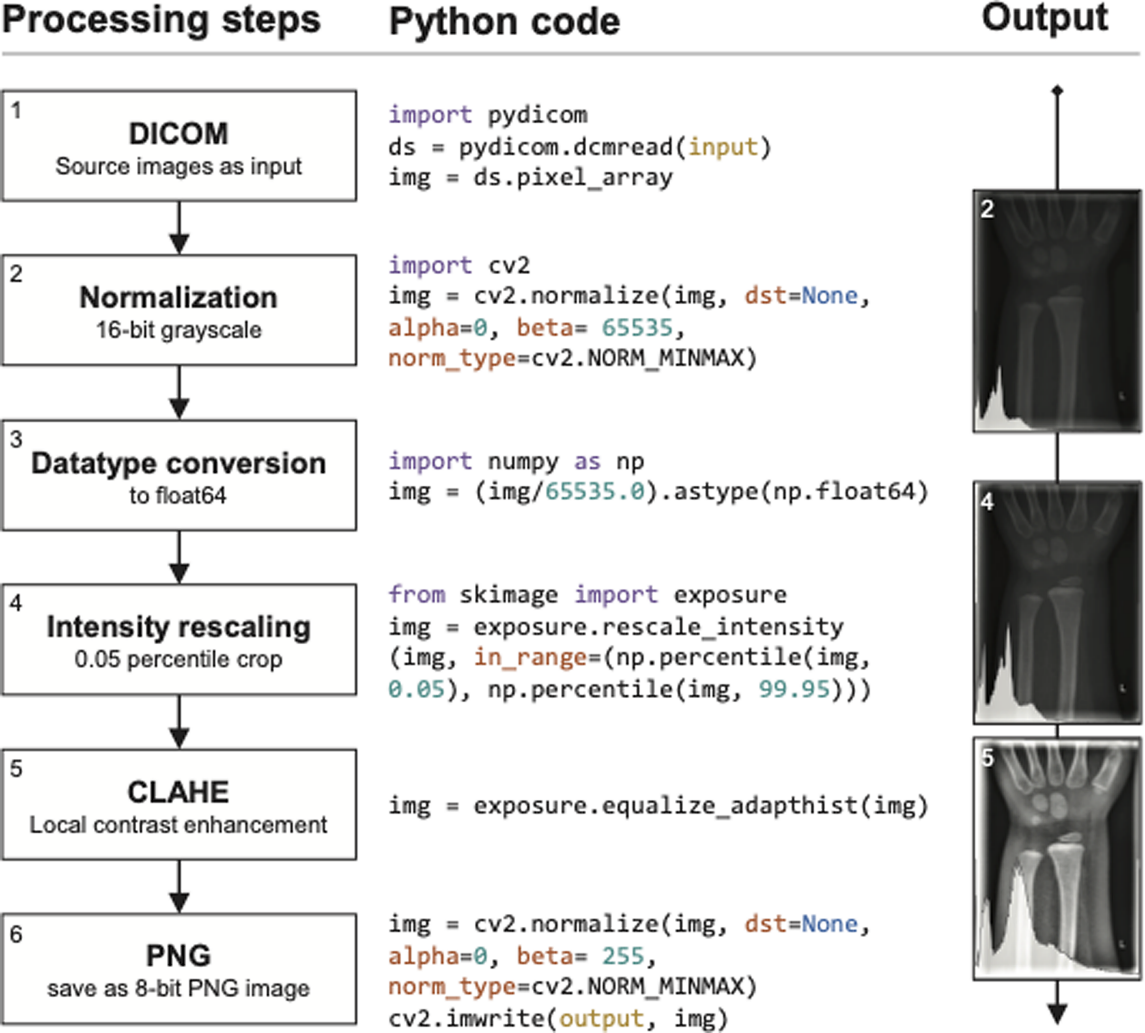
Image processing steps. DICOM images served as input, 8-bit PNG images as output used in training neural networks and human expert rating.

We retrieved the DICOM studies from PACS and converted them to portable network graphics (PNG) format. DICOM images featured 12-bit or 16-bit in grayscale values and different image dimensions. We converted the DICOMs to 16-bit PNGs through the “pydicom” and “cv2” packages in Python, initially normalizing their underlying grayscale spectrum to 16-bit and keeping original pixel dimensions. Furthermore, the images were post-processed with the “exposure” module of the Python “scikit-image” package. The 16-bit inputs were converted to float64 by dividing their grayscales through 65,535. Afterwards intensity rescaling was applied, cropping the lower and upper 0.05th percentiles of the image histograms. On top of that, local contrast enhancement “exposure_adapthist” with standard settings was applied. The float64 image data was converted to 8-bit outputs and saved to disk for further analyses. Throughout the described steps, images kept their original height and width. The processed images were re-scaled to the required input sizes on-the-fly while training. Figure 2 shows the image processing steps.

### CNN models and training

Convolutional neural networks (CNN) are used in computer vision for complex pattern recognition and regularly achieve excellent results in various tasks. CNN input data is fed into the multi-layer model and output after being processed *via* hidden layers. It consists of convolutional layers, pooling layers and fully connected layers, which are formed into a CNN in a certain manner (12).

We arbitrarily selected three common CNN models that we were able to train on consumer hardware available at the authors' institutions, namely EfficientNet-B4, ResNet-152, and VGG16. We could not assess the performance of the potentially more powerful EfficientNet-B7 due to graphics memory restrictions. All three CNN models were pre-trained on the comprehensive Imagenet dataset, meaning that we used the principles of transfer learning in our experiments. With transfer learning only the last few network layers important for predicting an image are trained, whereas the more basic lower levels of the CNN are not altered. With this approach, training times are substantially reduced (8). “Very Deep Convolutional Networks for Large-Scale Image Recognition (VGG16)” was published in 2014 and is still a widely used CNN. Due to the very high number of parameters, training is time-consuming compared to newer networks. Since 2015, deeper networks have been deployed that can be grouped together as ResNet CNNs while exhibiting lower complexity than previous CNNs. One of the latest state-of-the-art CNNs, EfficientNet, which was published in 2019, stands out for an even higher accuracy and faster processing on publicly available comparative data sets. By utilizing a compound scaling method for width, depth and resolution (EfficientNet-B0–EfficientNet-B7), even higher accuracies have been achieved (13–15).

We trained the above mentioned neural networks on a Linux personal computer, including a graphics card with 11 Gigabytes of memory. We used the Fastai Python library to load and train the CNN models. Images were fed into the training process after the previously described image processing steps. Furthermore, we have applied data augmentation to make models more robust to the data variability. The applied data augmentation is a fastAI module “aug_transforms” that is applying following augmentation on each image: image is randomly flipped with probability p=0.5, randomly rotated to a maximum of $10^{\circ }$ with a probability of p=0.75, the brightness and contrast are enhanced by a factor 0.2 with a probability of p=0.75, and last, but not least, we are zooming into a random part of the image (zoom ratio is between 1.0 and 1.1) with a probability of 0.75.

All models were trained for 100 epochs. After each epoch, the model was evaluated on the validation set where the combination of the best-performing weights was saved. Namely, the experiments have shown that models achieved the best validation score around epoch 40, which means that 100 epoch was more than enough to obtain the best possible result for given hyperparameters. Furthermore, the optimizer was Adam with a learning rate α=0.0001.

### Test interpretation by humans

The radiographs were interpreted in darkened reading rooms on radiology workstations with color-calibrated radiology monitors RX240, RX440, or RX650 (Eizo, Ishikawa, Japan). Human raters assessed the radiographs in full pixel resolution. The studies were hosted and displayed *via* the Supervisely artificial intelligence online platform (Deep Systems LLC, Moscow, Russia). Seven human expert raters with varying degrees of professional experience in interpreting pediatric trauma radiographs assessed the test set and were asked to carry out a binary classification as to whether a distal radius fracture was present or not.

### Test metrics

We calculated commonly accepted and widely used performance parameters for artificial intelligence including sensitivity (TPR), specificity (TNR), *F*1-score (*F*1) and Youden's Index (*J*) (16). The foundation of the test metrics is the confusion matrix in which positive and negative predictions are compared to true conditions in the way of a 2 × 2 cross table containing true positives (TP), false positives (FP), true negatives (TN) and false negatives (FN):

$$\mathrm{Sensitivity}:\;\mathrm{TPR}=\frac{\mathrm{TP}}{\mathrm{TP}+\mathrm{FN}}$$

$$\mathrm{Specificity}:\;\mathrm{TNR}=\frac{\mathrm{TN}}{\mathrm{TN}+\mathrm{FP}}$$

$$\mathrm{Positive}\;\mathrm{predictive}\;\mathrm{value}:\;\mathrm{PPV}=\frac{\mathrm{TP}}{\mathrm{TP}+\mathrm{FP}}$$

$$\mathrm{Negative}\;\mathrm{predictive}\;\mathrm{value}:\;\mathrm{NPV}=\frac{\mathrm{TN}}{\mathrm{TN}+\mathrm{FN}}$$

$$\mathrm{Accuracy}:\;\mathrm{ACC}=\frac{\mathrm{TP}+\mathrm{TN}}{\mathrm{TP}+\mathrm{TN}+\mathrm{FP}+\mathrm{FN}}$$

$$F1\text{-}\mathrm{Score}:\;F1=\frac{2\mathrm{TP}}{2\mathrm{TP}+\mathrm{FP}+\mathrm{FN}}$$

$$\mathrm{Youde}n^{\prime }s\;\mathrm{Index}:\;J=\mathrm{TPR}-(1-\mathrm{TNR})$$

We generated heatmaps with the Grad-CAM method to highlight the image regions that caused to a certain classification (17).

### Statistical analyses

We performed statistical calculations with IBM SPSS Statistics version 21 (IBM, Armonk, New York, United States) including descriptive statistics and non-parametric tests. McNemar's test served as a comparison parameter between CNN models and human raters for paired nominal data, constructed from the respective correct and incorrect ratings. To correlate the prediction of human raters and computer vision with patients' age, Pearson correlation coefficient was calculated. ROC analysis was conducted for binary fracture classification (yes or no) in computer vision models and all human experts individually. A ROC curve with continuous distribution was also calculated, using the CNN models' output probabilities, and expanded by the mean of the human expert ratings. To compare areas under the ROC curves the DeLong method (18) was performed, using MedCalc Software Version 19.6.4 (Ostend, Belgium). *P* values below 0.05 were assumed to be statistically significant.

### Ethical statement

The institutions ethics committee gave an affirmative vote for the retrospective data analyses (No. 31-108 ex 18/19). Informed patient or legal representative consent was waived. All experiments were performed in accordance with the local legal regulations and the declaration of Helsinki.

## Results

**Figure 3:**
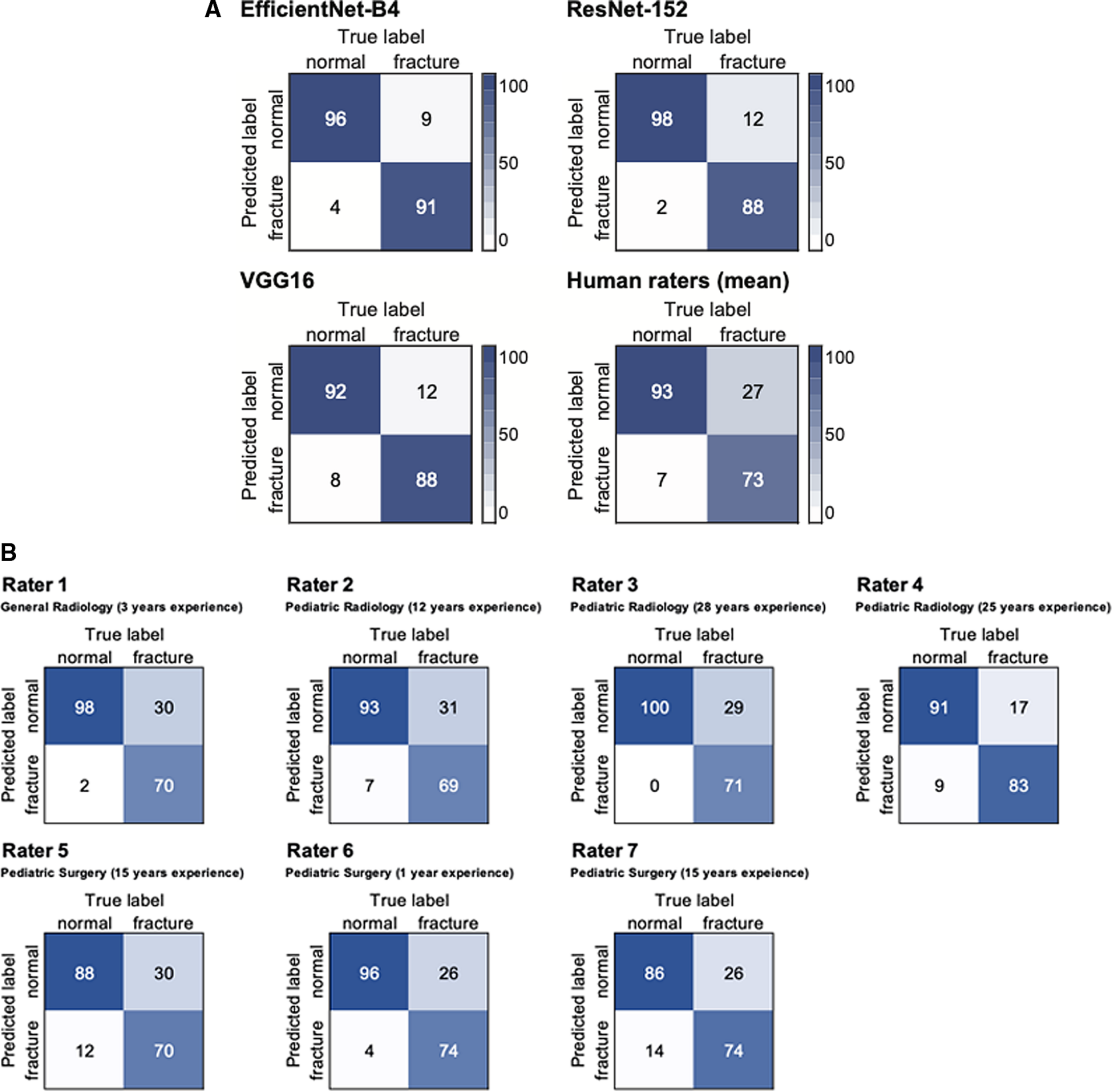
Confusion matrices of the test dataset (*n* = 200) for CNN models and the mean rating of human experts (**A**) as well as individual performances (**B**).

Pairwise McNemar comparisons showed significantly better performances of EfficientNet-B4 and ResNet-152 compared to all reference human readers (compare Table 1) VGG16 overperformed 4 raters out of 7 (*P*-values, 0.002–0.050), while statistical analysis did not show significant differences to Rater 3, Rater 4, and Rater 6 (*P* = 0.163, 0.418, and 0.134, respectively). Comparing the performance of radiologists (Rater 1–4) and pediatric surgeons (Rater 5–7) McNemar test showed statistically significant differences between Rater 4 and Rater 5 (*P* = 0.018), Rater 4 and Rater 7 (*P* = 0.034), as well as Rater 3 and Rater 5 (*P* = 0.026). Otherwise, no significant differences could be observed, comparing reference readers. Confusion matrices of the models and of human readers are shown in Figure 3.

**Table 1:** Binary classifications for CNN models and human experts in test dataset (*n* = 200). AUC with 95% confidence intervals in parentheses); *P*Δ, *P*-values for pairwise comparison of ROC curves, using the DeLong method; TPR, sensitivity; TNR, specificity; PPV, positive predictive value; NPV, negative predictive value; *F*1, *F*1-score; P*, McNemars test *P*-values with EfficientNet-B4 as reference.

	AUC (95% CI)	*P*Δ	TPR	TNR	PPV	NPV	ACC	*F*1	P*
**CNN**									
EfficientNet-B4	0.935 (0.895–0.975)	–	91.0	96.0	95.8	91.4	93.5	0.93	Reference
ResNet-152	0.930 (0.889–0.971)	0.781	88.0	98.0	97.8	89.1	93.0	0.93	0.286
VGG16	0.900 (0.852–0.948)	0.144	88.0	92.0	91.7	88.5	90.0	0.90	0.210
**Human Experts**									
Rater 1	0.840 (0.781–0.899)	0.001	70.0	98.0	97.2	76.6	84.0	0.81	0.002
Rater 2	0.810 (0.747–0.873)	0.001	69.0	93.0	90.8	75.0	81.0	0.78	0.001
Rater 3	0.855 (0.799–0.911)	0.001	71.0	100.0	100.0	77.5	85.5	0.83	0.003
Rater 4	0.870 (0.816–0.924)	0.018	83.0	91.0	90.2	84.3	87.0	0.86	0.031
Rater 5	0.790 (0.725–0.855)	0.001	70.0	88.0	85.4	74.6	79.0	0.77	0.001
Rater 6	0.850 (0.793–0.907)	0.001	74.0	96.0	94.9	78.7	85.0	0.83	0.003
Rater 7	0.800 (0.736–0.864)	0.001	74.0	86.0	84.1	76.8	80.0	0.79	0.001

EfficientNet-B4 achieved the highest sensitivity, NPV, and accuracy, whereas ResNet-152 had a higher specificity and a higher PPV. VGG16 was outperformed by the other CNN models, showing lower values in all metrics. The calculated F1-score as an overall measure for accurate binary classification was 0.93 for EfficientNet-B4 and ResNet-152, and 0.9 for VGG16. Human raters had sensitivities and specificities ranging from 69% to 83% and 86% to 100%, respectively. Related F1 scores were set between 0.77 and 0.86. Detailed results of binary classification are given in Table 1. While for VGG16, Rater 6 and Rater 7 the majority of false positives were assigned to the subgroup of the bone age dataset (63%, 100% and 85%, respectively), Rater 1, Rater 2 and Rater 4 have predominantly suspected fractures in inconspicuous wrist x-rays (100%, 57% and 78%, respectively). False positives of EfficientNet-B4, ResNet-152, and Rater 5 were equally distributed between the two subgroups (50% each). Rater 3 did not produce false positives.

**Figure 4:**
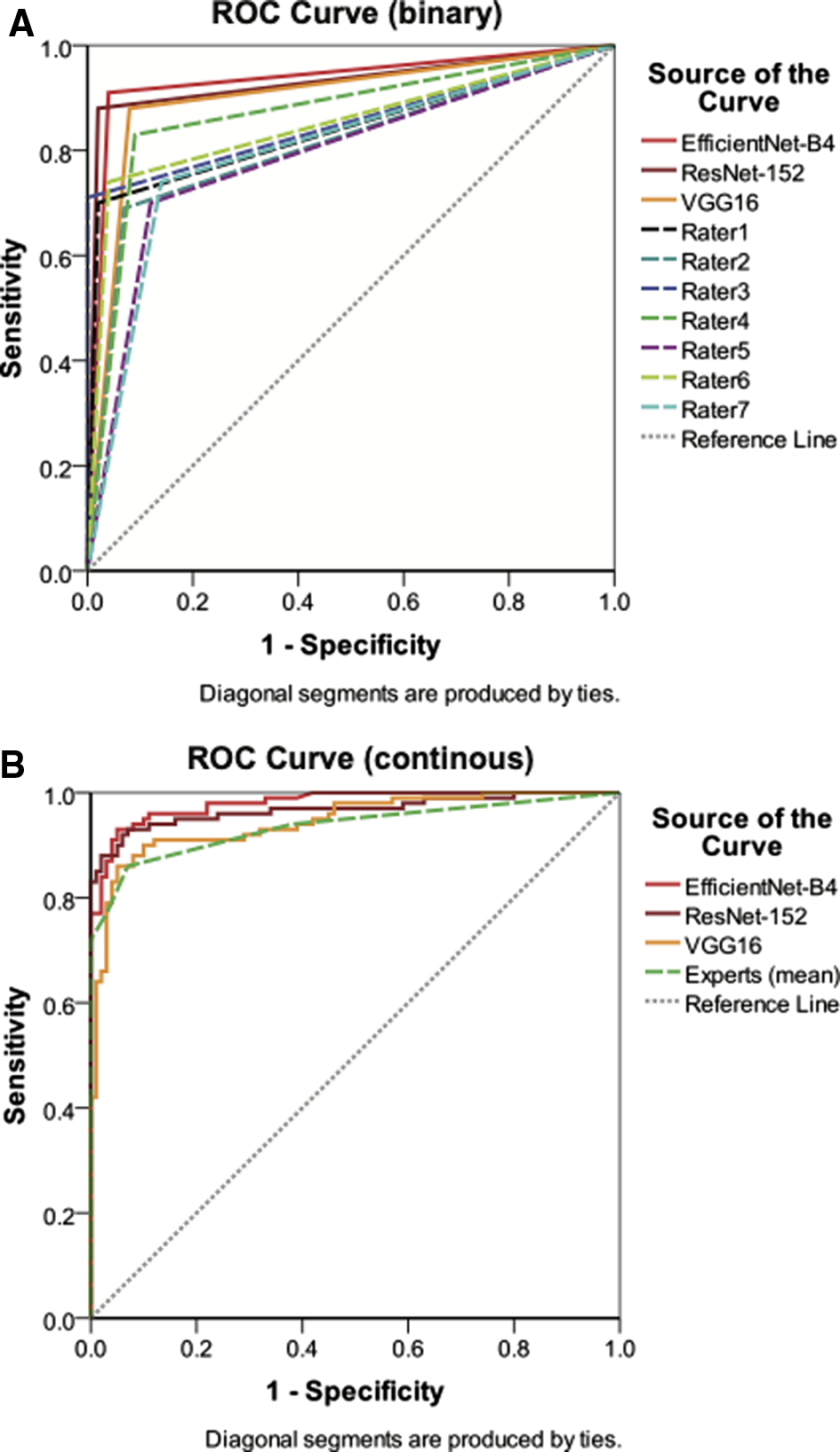
Binary (**A**) and continuous (**B**) data ROC analysis of diagnostic performance for CNN models and human experts in the test dataset (*n* = 200).

Continuous data ROC analysis of the three CNN model predictions achieved AUCs between 0.945 and 0.980 (CI 95%: 0.914–0.995) on the previously unseen test set (*n* = 200), with EfficientNet-B4 demonstrating the highest value. The AUC of human experts' results, when averaged, were significantly lower than EfficientNet-B4 (*P* = 0.009), based on the method by DeLong. Pairwise AUC comparison of human raters with ResNet-152 and VGG16 respectively did not yield significant differences (*P*-values > 0.05). Detailed results of ROC analysis for CNN based models and human raters are illustrated in Table 2. ROC curves are displayed in Figure 4.

**Table 2:** Continuous data ROC analysis of CNN model predictions in percent and averaged human expert ratings for detection of pediatric distal radius torus fractures in PA wrist radiographs. AUC, area under the curve; 95% CI, confidence interval; *J*, Youden's Index; P*, *P*-values relate to the comparison with EfficientNet-B4, using the DeLong method.

	AUC	95%CI	*J*	P*
EfficientNet-B4	0.980	0.966–0.995	0.88	Reference
ResNet-152	0.968	0.943–0.993	0.86	0.309
VGG16	0.945	0.914–0.975	0.81	0.018
Human experts (mean)	0.935	0.899–0.972	0.79	0.009

**Figure 5:**
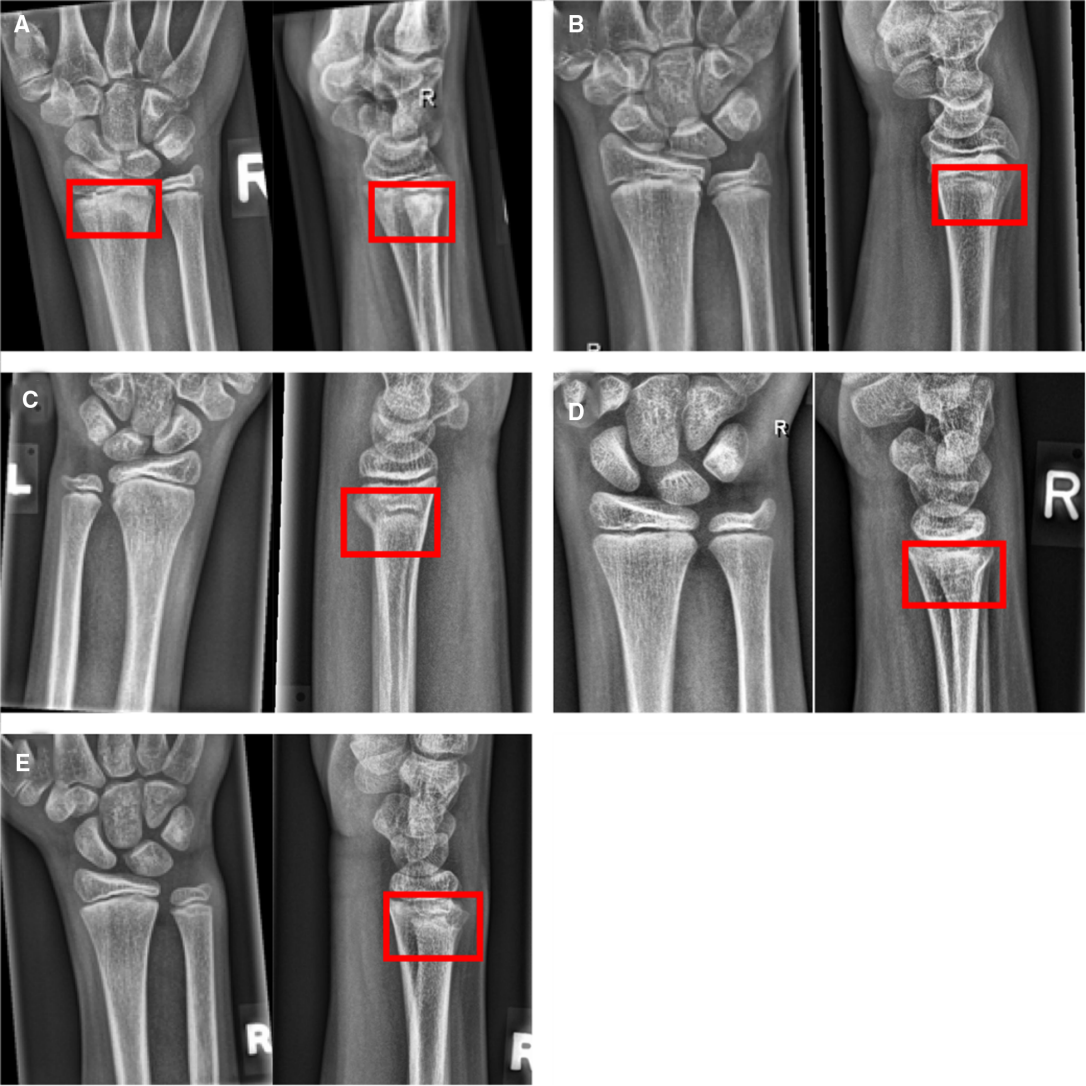
Example studies featuring the most common false negative cases. Lateral projections, if available, have been added to the figure to provide evidence for the presence of the fracture, but have not been part of the test set. (**A**) Distal radius fracture (red boxes) missed by all CNN models, but seen by 3 of 7 human raters. (**B**) Subtle dorsal distal radius fracture, missed by all CNN models and human experts. (**C–E**) Incomplete distal radius fractures missed by all raters but detected by the three CNN models.

**Figure 6:**
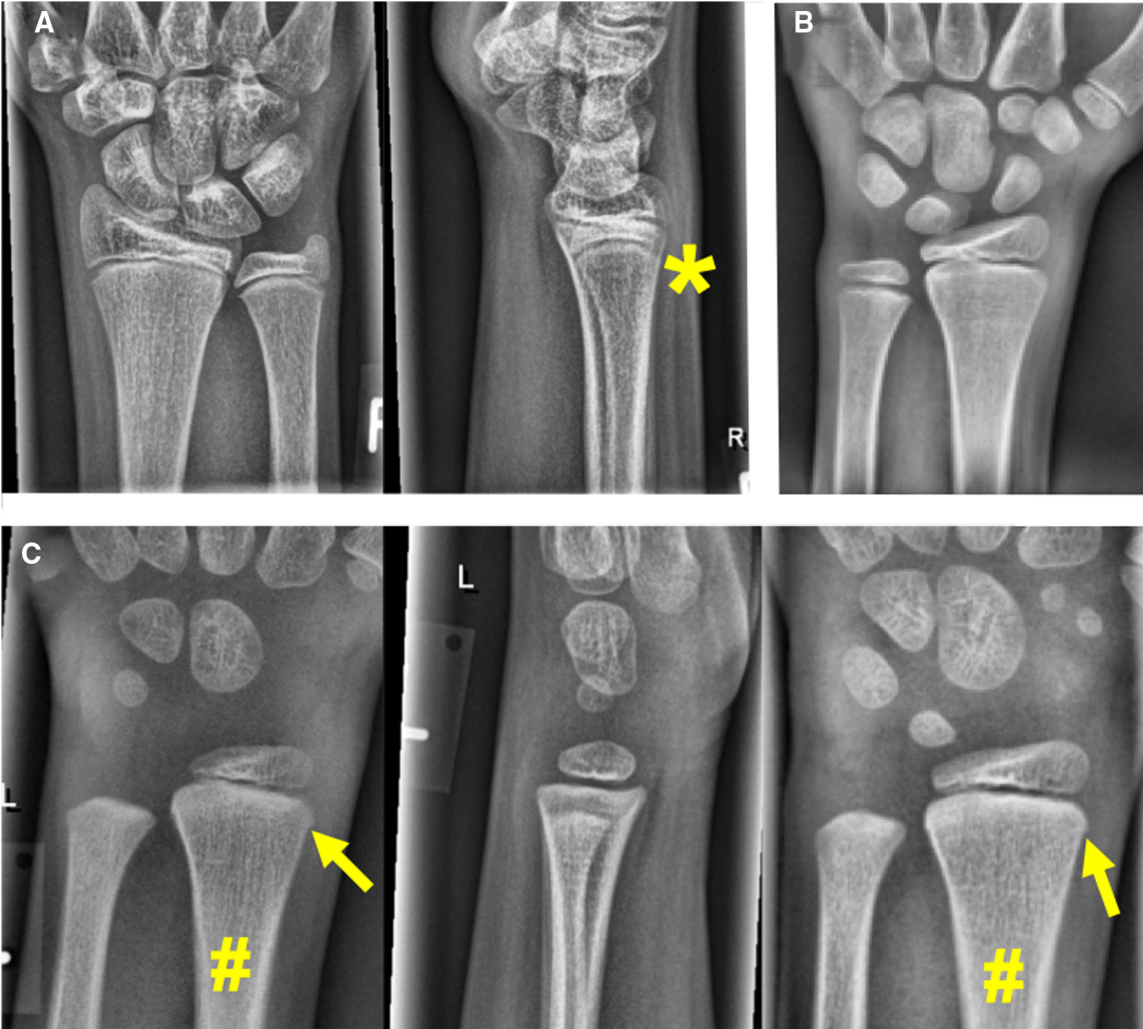
Example studies featuring the most common false positive cases. (**A**) EfficientNet-B4 and ResNet-152 predicted a fracture in a case categorized as negative by the reference radiologists. There is subtlest buckling next to the asterisk (*), so a fracture cannot be ultimately ruled out. (**B**) VGG16 and 3 of 7 human experts predicted a fracture in this asymptomatic patient's cropped hand x-ray (these studies do not include lateral projections). (**C**) A negative case rated positive for fracture by 3 of 7 human experts. The PA projection on the right was taken two years later. The arrows point at cortical irregularities commonly encountered in the pediatric distal radius, which are atypical for a fracture. The plus symbol (+) marks a discrete thickening of the distal radius, which is not moving away from the growth plate in the follow-up on the right. Therefore, a healed fracture is also unlikely in that location.

For all three AI models as well as for all human raters, a negative correlation of prediction and patient age, with values between -0.207 and −0.102, was observed, although only the results for VGG16 and human raters turned out to be statistically significant (*P*-values of 0.033 and 0.003, respectively). Example studies of false positives and false negatives are featured in Figures 5, 6.

## Discussion

We assessed three selected computer vision models in detecting acute distal radius torus fractures based on single projection (PA) pediatric wrist radiographs, comparing them to radiologists and pediatric surgeons. AI models outperformed human readers, who combined 99 cumulative years of experience in pediatric trauma radiography.

Results of ROC comparisons with continuous data as well as binary classifications showed statistically significant better performances of AI models, primarily EfficientNet-B4, than reference radiologists and pediatric surgeons. The calculated F1 score was also higher for CNN based models (0.90–0.93) than for human experts (0.77–0.86), confirming the more accurate fracture detection in the test set (*n* = 200). The substantially higher sensitivity of computer vision is particularly noteworthy, as fractures that are apparently more difficult for human experts to detect have also been correctly identified. Comparing the results among human raters, the highest F1 scores and AUCs were achieved by experienced pediatric radiologists although only Rater 4 obtained significantly better results, compared to reference pediatric surgeons (Rater 5 and 7).

Previous studies showed the potential of CNN based deep learning models assisting clinicians in the emergency department by detecting fractures on x-ray images accurately (2, 19–21). Thian et al. (22) have trained deep learning models for unspecifically detecting different kinds of fractures on not only pediatric but also adult wrist radiographs in PA and lateral projections. Since the appearance of pediatric and adult fractures differ substantially due to the presence of growth plates, from our point of view models exclusively designed for pediatric patients are obligatory (23). In contrast to (24), we used a larger training data set. Although they achieved similar accuracy for the detection of distal radius fractures in posteroanterior radiographs, we believe that our results are even more convincing because we only included torus fractures in our study which are more difficult to detect due to the discrete changes in plain radiographs and hence the expected accuracy for correct predictions were lower by nature. To underline the models' results and to put this aspect in perspective, we have validated the AI models' performances vs. human experts, while this comparison is missing in the referred study.

Our goal for the CNN based models was to detect the very common changes caused by torus fractures of the distal radius. These changes can be subtle and are also often overlooked by human readers (25). To the best of our knowledge AI-assisted detection of pediatric torus fractures has not been investigated and reducing the number of radiographs obtained to a single PA wrist x-ray in pediatric patients has not been approached yet. The results of our study underline the potential of deep learning algorithms in supporting medical experts in the management of pediatric wrist trauma. Adequate interpretation of the carpal bones and metacarpal bases requires PA or oblique views (26, 27), while lateral projections are typically insufficient to diagnose fractures located in these areas. Therefore, we believe that it is unavoidable to acquire at least the PA view of the wrist. We could imagine a computer vision system, when trained on larger amounts of radiographs, being able to rule-out fractures of the distal radius in PA projections only in a high percentage of cases. If directly implemented into a radiography workflow, performing the lateral projection might not be necessary in lots of pediatric wrist trauma cases, in order to reduce the radiation doses, avoid pain and stress caused by positioning, and also in terms of time savings for radiological technologists. Still, training such a model would require multitudes of the cases available in the current study.

Differentiating torus fractures from more severe wrist fractures should be investigated in future studies as it is highly important for adequate patient management (28). Displaced or complex fractures require a different management extending beyond cast immobilization, and often requiring surgical intervention (29).

As we assembled the negative test set (representing images without fractures) of hand radiographs for bone age determination and wrist x-rays to equal parts, we verified that there were no systematic differences between these sub-groups. Similar distributions of false positives were seen in both sub-groups by both CNN models and human raters. We consider that as a confirmation of labeling validity and working quality control procedures.

Some study limitations need be taken into consideration. This retrospective single center study had only access to a limited amount of data, and selection bias influencing the results cannot be ruled out. The ability to generalize to radiographs obtained at other institutions is yet unknown. A future large-scale multi-center study is believed to further improve the model's performance with the help of a more comprehensive training set. Further we arbitrarily selected three exemplary computer vision algorithms for this study. There might be models that perform better or worse on the current data. The underlying dataset was created and validated with the help of additionally obtained projections, follow-up examinations and further CT and MRI examinations (in limited number where available). The group of radiographs with normal appearance was also compiled carefully by using the second orthogonally-oriented projection and clinical data, but due to the lack of follow-up examinations in most cases, occult fractures cannot be ruled out with absolute certainty. By excluding all ambiguous cases in which we could not rule out a fracture with certainty, this risk was reduced to a minimum. Further, the distribution of false positives between wrist x-rays and cropped hand radiographs for bone age determination offers no indication of labeling errors. Lastly, we did not include other types of traumatic wrist injuries such as complete distal radius and ulna fractures. This downsized the training data set but also let us focus on subtle but common traumatic changes of the pediatric wrist.

There is still an issue with AI in medicine, since CNNs are regarded black-boxes with difficulties in explaining their underlying decision-making. According to Guidotti et al. (30), a possible way to get insight into the black-box model is to explain outcomes. In classification models this means to find the most important regions of interest that contributed to the final decision. For that purpose, we have used Grad-CAM class activation maps, proposed by Selvaraju et al. (17), “highlighting” the most relevant pixels in the images. The Grad-CAM is an enhanced version of Zhou et al. (31) that can work on any CNN without modifying the CNN. The method's idea is to backpropagate the weights from the end of the model towards the last convolutional layer of the model. The backpropagated gradients represent pondering factors to the feature maps of the last convolutional layer.

**Figure 7:**
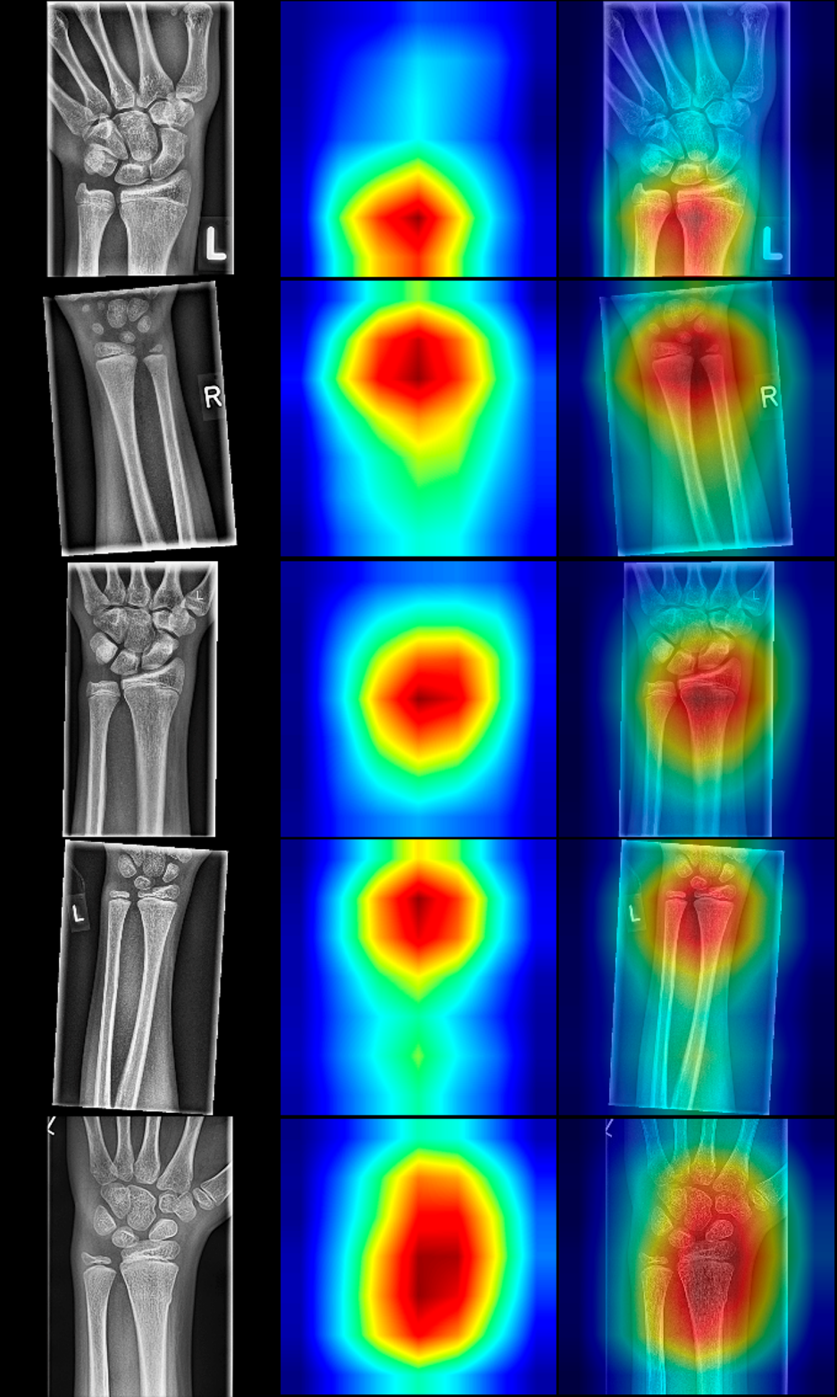
Random selection of radiographs positive for fracture with resNet-152 class activation maps (CAMs). The left column displays the source images, the middle column illustrates CAMs, and the right depicts fusions of source and sample images. Note that the classes are activated in the distal forearm region, even though the fields of view differ in the presented radiographs.

Upscaled summed feature maps to the input image size result in an attention heatmap that marks the regions that contributed most towards the final outcome of the CNN. This way, we can inspect why CNN predicted a particular result and explain our models. The resulted heatmaps are presented in Figure 7 and are showing that the focus of the CNNs is on the distal forearm region, which is precisely the region where the radiologist focuses during the fracture detection. Therefore, the CNNs mimic the radiologist by sharing the same attention field during the decision-making process.

In conclusion, we developed three CNN based deep learning models for the detection of distal radius torus fractures on single projection PA oriented radiographs. In the test performed, the models not only demonstrated accurate fracture detection, but also outperformed experienced radiologists and pediatric surgeons.

## Data Availability

The raw data supporting the conclusions of this article will be made available by the authors, without undue reservation.
